# Transactions of Chicago Medico-Historical Society

**Published:** 1874-06-01

**Authors:** Chas. W. Earle

**Affiliations:** Secretary


### TRANSACTIONS OF CHICAGO MEDICO-HISTORICAL SOCIETY.

### CONSTITUTION AND BY-LAWS.

AT a meeting held at the office of Dr. N. S. Davis, April 21st, 1874, composed of physicians representing the general profession, and the various colleges and hospitals in the city, Dr. Alex. Fisher was called to the Chair, and Dr. J. N. Hyde to act as Secretary.

On motion of Dr. Hay, seconded by Dr. Jackson, it was voted that a committee of five be appointed by the Chair, who should be empowered to draft the Constitution of an organization for the purpose of collecting and preserving the archives of the profession, and of registering the names and addresses of its legitimate members.

The Chair appointed as members of the Committee Drs. T. D. Fitch, Bridge, N. S. Davis, Bevan, and Hay.

At a subsequent meeting, held April 28th, 1874, in the Club Rooms of the Tremont House, the Committee submitted their report. After due deliberation, the following was declared to be the

### CONSTITUTION OF THE SOCIETY.

ART. I.—This Association shall be called "THE CHICAGO MEDICO-HISTORICAL SOCIETY."

ART. II.—Its objects shall be to discover, procure and preserve, whatever may relate to the medical history of Chicago and vicinity, and the publication of such information as may be from time to time determined upon.

ART. III.—It shall consist of, at first, not less than twenty-five members. Candidates for membership shall be nominated by the Committee on Publication, at a regular or special meeting; and at a subsequent meeting they may, on ballot, be elected by a three-fourths vote of all members present; *provided*, the first election of members shall be by the general meeting of the profession at which this organization is effected.

ART. IV.—Members may be suspended or expelled on charges of negligence of duty or other misconduct, preferred at a stated meeting, and being within five days thereafter communicated to the accused by the Secretary, at a subsequent stated meeting, by a two-thirds vote.

ART. V.—The officers of the Society shall consist of a President, a Vice-President, a Secretary, a Treasurer, a Diarist, and an Editor. There shall also be a Standing Committee on Publication, to consist of the President and Editor *ex officio*, and three elected members. These officers (excepting the Editor and Committee on Publication, whose duties are hereinafter designated) shall perform the duties usually appertaining to their respective positions. They (excepting the Editor) shall be elected at the anniversary meeting. The Editor shall hold office indefinitely; but a new election may be ordered by a majority vote, at the anniversary meeting, or at any stated meeting, on the requisition of the Committee on Publication, or of any five regular members of the Society, notice of such requisition having been given at a previous meeting, and by the Secretary, to each member.

ART. VI.—The Editor, with the advice and assistance of the Committee on Publication, shall prepare and publish "The Chicago Medical Register, etc.," and such other matter as the Society shall from time to time direct, under such regulations as may be recommended by the Committee on Publication and approved by the Society.

ART. VII.—The Committee on Publication, presided over by a Chairman of its own choice, shall assist the editor in the selection and preparation of material for publication, make all the necessary financial arrangements, and exercise such immediate control of the "Register" as it is inconvenient for the Society as a whole to exercise; but shall at no time admit or exclude from the "Register" the name of any practitioner whose claim to admission, or the justice of whose exclusion may be open to any question of doubt, except in obedience to the action of the Society, to which all questions of this nature shall be submitted at its several meetings. It shall attend to the keeping of the books, etc., appertaining to irregular practitioners, discharge the duties of the "Biographical Library," and "Portraiture Committees," and act as a Committee on Nominations. It shall report its proceedings to the Society at such times as the former may deem expedient, or the latter may order, and be in all things subject to the control of the Society.

The election under the Constitution resulted as follows:

Dr. R. C. Hamill, *President;* Dr. D. B. Trimble, *Vice-President;* Dr. A. R. Jackson, *Editor;* Drs. Bevan, Owen and Bridge, *Publishing Committee;* Dr. Wickersham, *Diarist;* Dr. Chas. W. Earle, *Secretary;* Dr. R. G. Bogue, *Treasurer.*

On motion, it was voted that a committee of three be appointed by the Chair to propose By-Laws for the Society.

The Chair appointed Drs. E. Ingals, Bartlett, and Dexter.

At a meeting held May 5th, the Committee on By-Laws submitted their report, and the Society, after careful consideration, declared the following to be the

### BY-LAWS OF THE SOCIETY.

ART. I.—The Society shall hold stated meetings on the last Tuesdays of January, April, July and October of each year, at 8 o'clock P.M. The annual election of officers shall be at the April meeting; but should there be no quorum for such election, the meeting may be adjourned from time to time, as circumstances may require. Special meetings shall be called by the Secretary, on the requisition of any five members of the Society: and the object of such meetings shall be stated in the notice to members by the Secretary.

ART. II.—The Order of Business shall be, 1st, Roll-Call; 2d, Reading of Minutes; 3d, Report of Treasurer; 4th, Report of Diarist; 5th, Report of Committee on Publication; 6th, Report of Special Committees; 7th, Unfinished Business; 8th, Miscellaneous Business; 9th, Adjournment.

ART. III.—No one shall be admitted to membership in the Society who does not give satisfactory evidence of having received a diploma from some respectable medical college; and violations of the code of the American Medical Association shall be cause of rejection or expulsion.

ART. IV.—Any member who shall have omitted payment of dues for three months, or who shall have absented himself from three consecutive stated meetings, shall be declared by the President, at the next subsequent stated meeting, to have thereby forfeited his membership; *provided*, the Secretary shall have given notice to such member of his neglect, and its consequences, and the penalty is not remitted by vote of the Society. Permanent removal from the city may be decided by vote of the Society as equivalent to resignation.

ART. V.—Any funds necessary for the carrying on of the work of this Society shall be raised by regular and equal assessment of all the members, said assessment to be made by the Committee on Publications, subject to the approval of the Society.

The Society adjourned, to meet at its stated time, the last Tuesday in July, or subject to call by the Secretary, as provided in Art. I, By-Laws.

CHAS. W. EARLE, Secretary.

